# CXCR4-targeted PET/CT in early systemic sclerosis–associated interstitial lung disease: a prospective proof-of-concept study of in vivo inflammatory activity

**DOI:** 10.1007/s00259-026-08000-3

**Published:** 2026-06-16

**Authors:** Michael Gernert, Marc Schmalzing, Hannah Labinsky, Yingjun Zhi, Patrick-Pascal Strunz, Matthias Fröhlich, Philipp E. Hartrampf, Andreas K. Buck, Rudolf A. Werner, Sebastian E. Serfling

**Affiliations:** 1 Department of Internal Medicine 2, Rheumatology/Clinical Immunology, University Hospital Würzburg, Würzburg, Germany; 2 Department of Otorhinolaryngology, Head and Neck Surgery, University Hospital Würzburg, Würzburg, Germany; 3 Department of Nuclear Medicine, University Hospital Würzburg, Oberdürrbacher Str. 6, 97080 Würzburg, Germany; 4 The Russell H Morgan, Department of Radiology and Radiological Sciences, Johns Hopkins School of Medicine, Baltimore, MD USA; 5 Department of Nuclear Medicine, LMU University Hospital, LMU Munich, Munich, Germany

**Keywords:** Systemic sclerosis, Interstitial lung disease, Connective tissue disease, Positron emission tomography, [^68^Ga]Pentixafor, C-X-C motif chemokine receptor 4

## Abstract

**Introduction:**

Distinguishing active inflammatory or remodeling-associated processes from irreversible fibrosis in systemic sclerosis remains challenging using conventional imaging modalities. The tracer [^68^Ga]Pentixafor targets the C-X-C motif chemokine receptor 4 (CXCR4) which is expressed on leukocytes. Uptake of [^68^Ga]Pentixafor may provide complementary biological information on CXCR4-associated inflammatory and remodeling activity.

**Methods:**

This prospective cohort study included patients with early SSc (disease duration ≤ 5 years) and preexisting interstitial lung disease. All patients underwent [^68^Ga]Pentixafor-PET/CT imaging. Normalized [^68^Ga]Pentixafor uptake was quantified using the target-to-background ratio (TBR). Follow-up data on disease progression was collected. A control group comprised patients with giant cell arteritis.

**Results:**

Twelve patients with SSc were included. The highest [^68^Ga]Pentixafor uptake was observed in the hilar and mediastinal lymph nodes, in fibrotic lung regions, and in the left ventricular myocardium. In all three regions, a significantly higher [^68^Ga]Pentixafor uptake was measured in SSc compared to controls (median TBR of lymph nodes 3.5 [IQR 2.7 – 4.3] vs 1.6 [1.3 – 2.0] *p* < 0.0001; inferior lung lobes 2.3 [1.6 – 2.8] vs 0.8 [0.7 – 1.0], *p* < 0.0001; and left ventricle 2.0 [1.4 – 2.4] vs 1.3 [1.1 – 1.5], *p* = 0.0178). Patients who developed progressive lung disease during follow-up showed a higher [^68^Ga]Pentixafor uptake compared to non-progressive patients reaching statistical significance in the left ventricle (TBR of progressive vs non-progressive patients: 2.5 (2.1 – 3.3) vs 1.5 (1.3 – 2.1), *p* = 0.0283) and showing a numerical increase in fibrotic lung regions (2.8 (2.2 – 3.3) vs 1.7 (1.1 – 2.8), *p* = 0.1535).

**Conclusion:**

These findings provide proof-of-concept evidence that CXCR4-targeted PET/CT may visualize biologically active inflammatory and/or remodeling processes in early-stage SSc-ILD.

**Supplementary Information:**

The online version contains supplementary material available at https://doi.org/10.1007/s00259-026-08000-3.

## Introduction

Systemic sclerosis (SSc) is characterized by different pathophysiologic mechanisms including vasculopathy, inflammation, and fibrosis [1]. Inflammation can be treated by immunosuppression but, if not addressed properly, may progress to irreversible and difficult-to-treat fibrosis. Early identification of inflammation therefore represents an unmet need in the routine clinical care with SSc. Persistent inflammatory activity despite immunosuppressive treatment may indicate the need for treatment escalation in selected patients; however, current imaging approaches provide limited information on ongoing biological activity. However, conventional CT imaging does not reliably differentiate between active inflammation (alveolitis) and early microfibrosis in the lung. Functional imaging may provide additional information to identify active inflammatory processes. [^18^F]FDG-PET/CT can detect inflammatory activity in the lungs of patients with SSc [2, 3], by reflecting glucose metabolism rather than specific leukocyte infiltration. Therefore, [^18^F]-FDG uptake is not specific for inflammation. Moreover, [^18^F]-FDG uptake is influenced by blood glucose levels [4], systemic metabolic conditions [5], and therapeutic interventions [6], which may limit its interpretability in patients with chronic diseases such as SSc.

In contrast, C-X-C motif chemokine receptor 4 (CXCR4) is abundantly expressed on leukocytes [7] and plays an important role in inflammatory cell trafficking [8]. CXCR4 can be targeted by the PET tracer [^68^Ga]Pentixafor. Hence, [^68^Ga]Pentixafor uptake is considered to reflect the presence of CXCR4-expressing inflammatory cells and may serve as an imaging surrogate of inflammatory activity in various tissues [9, 10]. Stromal cell-derived factor-1 (SDF-1; also referred to as CXCL12) is the natural ligand binding to CXCR4^8^.

CXCR4-targeted PET imaging has previously been shown to detect inflammatory activity and predict treatment response in idiopathic pulmonary fibrosis [11]. More recently, CXCR4-directed imaging has also been explored in systemic sclerosis-associated interstitial lung disease using alternative tracer [12], supporting the biological relevance of this pathway. The CXCL12/CXCR4 axis plays a central role in inflammatory cell recruitment and has been implicated in the pathogenesis of pulmonary fibrosis [13].

The aim of this prospective pilot study was to evaluate the feasibility of CXCR4-targeted PET/CT for visualizing CXCR4-associated biological activity in patients with early-stage SSc-ILD and to explore associations between tracer uptake and subsequent organ disease progression.

## Patients and methods

### Patients and controls

Inclusion criteria were fulfillment of the ACR/EULAR 2013 classification criteria for SSc, presence of interstitial lung disease (ILD) on computed tomography (CT), and age ≥ 18 years. To recruit patients with early/inflammatory SSc disease duration was limited to ≤ 5 years (from the onset of the first non-Raynaud symptom). Key exclusion criteria were previous autologous stem cell transplantation, pulmonary hypertension, and pregnancy or breastfeeding. Consecutive patients fulfilling all eligibility criteria were recruited in the outpatient department of the rheumatology of the University Hospital of Wuerzburg. Subgroups of the SSc cohort were identified according to status of Scl70 autoantibody positivity, according to use of immunosuppression, or according to disease activity assessed by the European scleroderma trials and research group (EUSTAR) index [14] (< 2.5 points = inactive and ≥ 2.5 points = active disease. Points are accumulated for worsening of skin fibrosis, presence of digital ulcers, a mRSS Score > 18 points, presence of tendon friction rubs, CRP > 1 mg/dl and DLCO < 70% of predicted).

## Ethics approval and consent to participate

The study was approved by the local ethics committee (reference number 296/21-me) and the Federal Office for Radiation Protection (BfS), and conducted in accordance with the Declaration of Helsinki. All patients provided written informed consent prior to imaging.

The study was retrospectively registered at ClinicalTrials.gov (NCT07212959; first posted on October 8, 2025). At the time of registration, patient enrollment had already commenced. No changes to the study protocol or endpoints were made after trial registration.

The control group consisted of patients with giant cell arteritis (GCA) who had participated in a separate CXCR4-PET/CT pilot study (NCT05604482) [15].

### Clinical and serological parameters

As part of clinical routine, the following parameters were assessed before and after CXCR4-PET/CT: modified Rodnan skin score (mRSS), serum troponin, N-terminal prohormone of brain natriuretic peptide (NT-pro BNP), transthoracic echocardiography, pulmonary function testing including diffusion capacity, and CT of the thorax. Cutaneous progression was defined as an increase of mRSS ≥ 10%. Pulmonary progression was defined analogous to the definition of progressive pulmonary fibrosis in clinical trials [16] by the presence of at least one of the following criteria: decline in forced vital capacity (FVC) ≥ 10%, decline in diffusion capacity of carbon monoxide (DLCO) ≥ 10%, or increase of fibrosis ≥ 10% of the initial affected lung area on CT.

Plasma levels of SDF-1 were measured by ELISA (Thermo Fisher, Frederick, MD) according to the manufacturer’s instructions.

### Radiotracer preparation

[^68^Ga]Pentixafor was prepared in accordance with good manufacturing practice using an automated synthesis module (Scintomics, Fürstenfeldbruck, Germany) and disposable single-use cassette kits (ABX, Radeberg, Germany), as described elsewhere [17]. Quality control was performed before clinical application according to institutional standard operating procedures. Peptide mass, administered activity, and specific activity of [^68^Ga]Pentixafor were recorded for each patient.

### CXCR4-PET/CT

CXCR4-directed PET/CT imaging was performed 60 min after intravenous administration of [^68^Ga]Pentixafor (146 ± 7 MBq). Whole-body imaging was performed from the skull vertex to mid-thigh using a hybrid PET/CT system (Biograph mCT 128, Siemens Healthineers, Erlangen, Germany). PET data were acquired in three-dimensional mode and reconstructed iteratively according to the manufacturer’s standard implementation without time-of-flight reconstruction. In brief, reconstruction was performed using a 200 × 200 matrix, 3 iterations, at least 21 subsets, and a 2-mm Gaussian filter. Low-dose CT was acquired for attenuation correction and anatomical localization using the following parameters: reference tube current, 35 mAs; minimum tube voltage, 100 kVp; minimum pitch, 0.8; rotation time, 0.5 s; and reconstructed axial slice thickness, 3.0 mm.

Image interpretation of CT, PET, and fused PET/CT datasets was performed by an experienced nuclear medicine physician with more than 10 years of expertise. For quantitative analysis, volumes of interest (VOIs) were manually placed over predefined target regions, including the superior and inferior lung lobes, the middle lung lobe, the left ventricular myocardium, and mediastinal and hilar lymph nodes.

### VOI placement and quantitative image analysis

Pulmonary VOI placement was adapted from a previously published ROI-based lung quantification approach^12^. To account for anatomical lung structures and enable lobar-based statistical analysis, the original 22-ROI scheme was modified into a VOI-based anatomically approximated allocation model.

A standardized pulmonary 22-volume-of-interest (VOI) sampling scheme was applied. Circular VOIs with a volume of 2 cm^3^ were placed in both lungs and subsequently assigned to anatomically approximated lobar regions for statistical analysis. Four VOIs were assigned to the right upper lobe and five VOIs to the left upper lobe, including one ventral lingular VOI. One ventral VOI was assigned to the right middle lobe. Six VOIs were assigned to the right lower lobe, consisting of three medial posterior and three basal posterior VOIs, and six VOIs were assigned to the left lower lobe, also consisting of three medial posterior and three basal posterior VOIs. This resulted in a total of 22 pulmonary VOIs per patient. The anatomical distribution of VOIs is summarized in Supplementary Table S1, and representative examples of VOI placement are provided in Supplementary Figure S3.

For lymph nodes and left ventricular myocardium, VOI size was adapted to the respective anatomical structure to avoid spillover from adjacent tissues and blood-pool activity. Three Myocardial VOIs were placed within the left ventricular myocardium while avoiding the ventricular blood pool. Lymph node VOIs were placed over visually identifiable hilar and mediastinal lymph nodes. For background correction, three VOIs were placed along the superior vena cava, and SUV_mean_ was used as blood-pool reference.

SUV_max_ and SUV_mean_ values were recorded for all target regions. For blood-pool normalization, three VOIs were placed intraluminally along the superior vena cava, and their average SUV_mean_ was used as the blood-pool reference. Target-to-background ratios were calculated as SUV_max_ of the respective target region divided by the averaged SUV_mean_ of the superior vena cava.

Respiratory gating was not performed. Therefore, pulmonary uptake measurements, particularly in the lower lung lobes, may have been affected by respiratory motion and partial-volume effects. Test–retest imaging was not performed in this prospective pilot study.

### Statistical analysis

Calculations were done with Prism (V10; GraphPad Software, Boston, MA). For continuous variables differences between paired groups were examined with Wilcoxon signed-rank tests and differences between unpaired groups with Mann–Whitney-U tests. For metrical variables differences between unpaired groups were calculated with Fisher’s exact tests. Differences were considered significant when two tailed p-values were less than 0.05 (*), less than 0.01 (**), and less than 0.001 (***). Excel (Microsoft, Redmond, WA) was used to collect the data. Figures were grouped with paint.net (V5; dotPDN LLC, Kirkland, WA).

## Results

### Study population and control group

Twelve patients with SSc were included of whom seven were female (58.3%). Their median age was 64.5 years (range 34–83) with a median disease duration of 2.0 years (range 1–5). Skin involvement was mild with a median mRSS of 3 points (range 0–6), but all SSc patients had lung involvement with nonspecific interstitial pneumonia (NSIP) pattern and a median FVC of 85.0% of predicted and a median DLCO of 47.5% of predicted. At the time of the [^68^Ga]Pentixafor-PET/CT, eight SSc patients (66.7%) received mycophenolate (MMF), two (16.7%) rituximab, and five (41.7%) received nintedanib, either as add-on or monotherapy. The median prospective follow-up was 9.2 months (range 5–18).

The control group comprised ten patients with GCA, 60.0% of whom were female with a median age of 66.9 years (range 61–79). Eight GCA patients (80.0%) were without immunosuppressive treatment, two had received prednisolone at the time of the [^68^Ga]Pentixafor-PET/CT. Characteristics of both groups are provided in Table 1.

**Table 1:** Characteristics of the study population and control group

Characteristics	SSc patients	Control group (GCA patients)
Female, *n* (%)	7/12 (58.3)	6/10 (60.0)
Age, median (range), years	64.5 (34–83)	66.9 (61–79)
Disease duration at the time of CXCR4-PET/CT, median (range), years	2.0 (1–5)	na
mRSS, median (range), points	3.0 (0–6)	na
Lung involvement/ILD, *n* (%)	12/12 (100.0)	0/10 (0.0)
Forced vital capacity, median (range), % of predicted	85.0 (52–110)	nd
Diffusion capacity of carbon monoxide, median (range), % of predicted	47.5 (35–91)	nd
Cardiac involvement^§^, *n* (%)	3/12 (25.0)	na
Smoking history ever, *n* (%):	5/12 (41.7)	1/10 (10.0)
Anti-Scl-70 antibody positivity, *n* (%)	8/12 (66.7)	na
C-reactive protein, median (range), mg/dl	0.3 (0.0–2.0)	6.3 (0.1–11.0)
Immunosuppressive treatment at the time of CXCR4-PET/CT, *n* (%)		
Mycophenolate	8/12 (66.7)	0/10 (0.0)
Rituximab	2/12 (16.7)	0/10 (0.0)
Prednisolone	0/12 (0.0)	2/10 (20.0)
None	2/12 (16.7)	8/10 (80.0)
Antifibrotic treatment with nintedanib, *n* (%)	5/12 (41.7)	0/10 (0.0)

*GCA* giant cell arteritis, *ILD* interstitial lung disease, *mRSS* modified Rodnan skin score, *nd* not done, *SSc* systemic sclerosis

^§^ i.e. high-sensitive troponin above upper limit of normal + myocardial edema or late enhancement in cardiac MRI

**Fig. 1:**
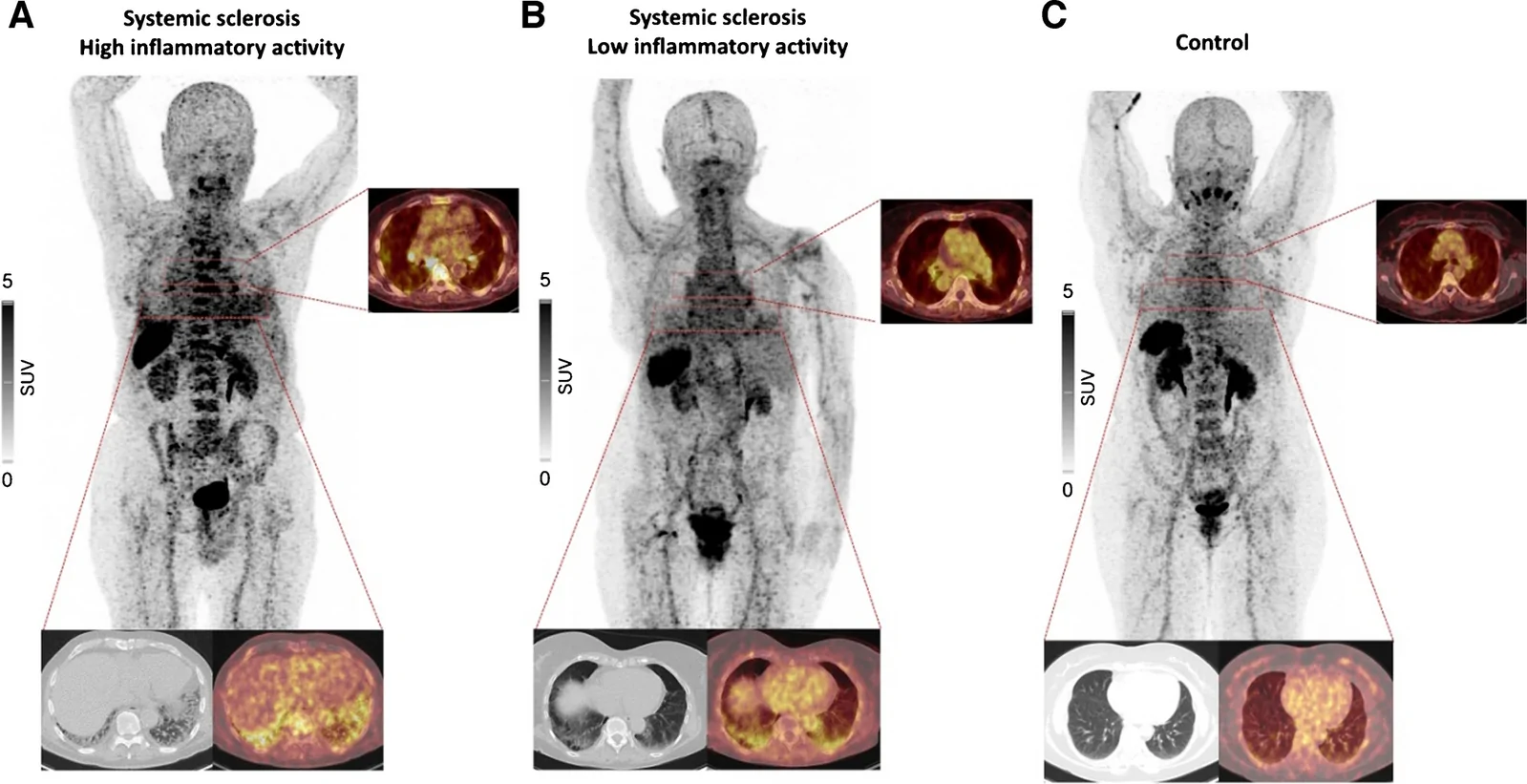
Representative CXCR4-PET/CT scans. CT-scan inserts show the hilar lymph nodes (right side) and the inferior lung parts. **A**) A systemic sclerosis patient with high inflammatory burden. Consecutive high [^68^Ga]Pentixafor uptake in the bone marrow of the spine and pelvis. **B**) A systemic sclerosis patient with low inflammatory activity, and C) CXCR4-PET/CT scan of a patient with giant cell arteritis serving as control for lung and hilar lymph node tissue

In patients with SSc, increased [^68^Ga]Pentixafor uptake was observed in CT-defined ILD-affected lung regions, hilar and mediastinal lymph nodes, the left ventricular myocardium, and the bone marrow. As expected, physiological tracer accumulation was consistently seen in the spleen. Representative [^68^Ga]Pentixafor-PET/CT images are shown in Fig. 1.

### Higher [^68^Ga]Pentixafor uptake in fibrotic versus non-fibrotic lung regions

**Fig. 2:**
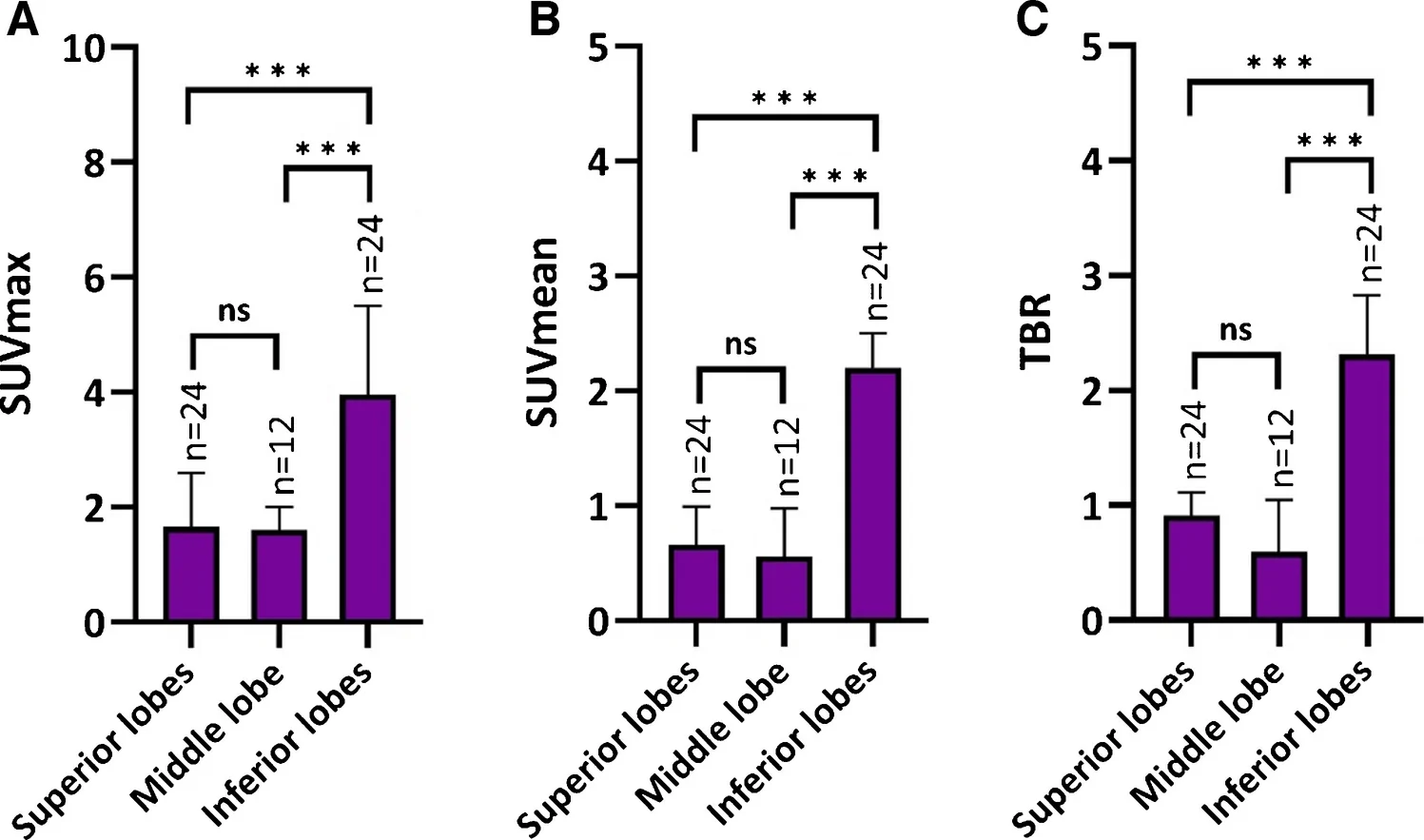
Intrapatient comparison of [^68^Ga]Pentixafor uptake in unaffected lung areas with fibrotic lung areas. **A**) SUVmax, **B**) SUVmean, and **C**) target-to-background ratio (TBR) with higher [^68^Ga]Pentixafor uptake in the inferior lung lobes, compared to the middle lobe and superior lobes. ns = not significant

Within the SSc cohort [^68^Ga]Pentixafor uptake was significantly higher in the inferior lung lobes, corresponding to areas affected by ILD in all patients compared with the middle lobe and superior lobes where no ILD was present (Fig. 2). TBR of the inferior lobes vs TBR of superior lobes was: 2.3 (IQR 1.6–2.8) vs 0.9 (0.5–1.1), *p* < 0.0001. SUVmax and SUVmean values were likewise significantly higher in the inferior lobes compared with the middle and superior lobes.

### Higher [^68^Ga]Pentixafor uptake in SSc patients compared to controls

**Fig. 3:**
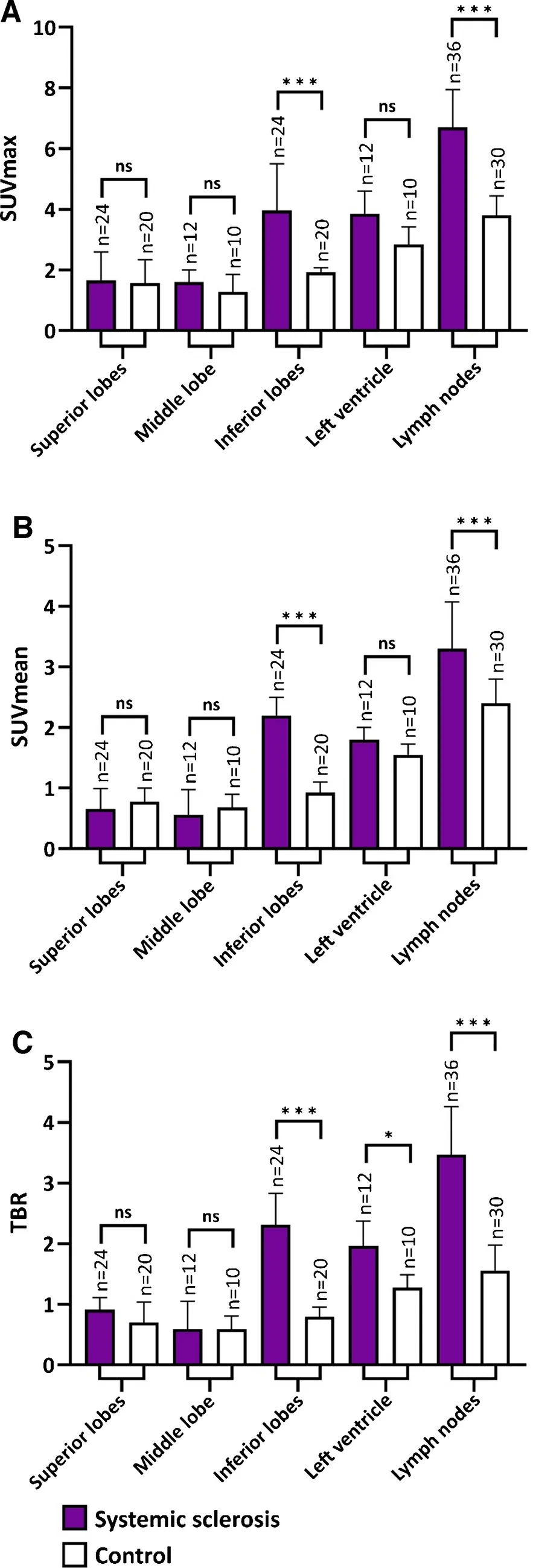
Comparison of [^68^Ga]Pentixafor uptake of different areas in systemic sclerosis patients versus controls. **A**) SUVmax, **B**) SUVmean, and **C**) target-to-background ratio (TBR) with consistent higher [^68^Ga]Pentixafor uptake in systemic sclerosis in the inferior lung lobes and lymph nodes. Superior lobes and inferior lobes include the right and left side, respectively. Lymph nodes include measurements of 3 regions: Left hilar, right hilar, and mediastinal lymph nodes. Purple bars: Systemic sclerosis; white bars: Control. *ns* not significant

Compared with controls, patients with SSc demonstrated a significantly higher [^68^Ga]Pentixafor uptake in three regions: the inferior lung lobes (TBR SSc 2.3 [IQR 1.6–2.8] vs TBR control 0.8 [0.7–1.0], *p* < 0.0001), in the left ventricle of the heart (2.0 [1.4–2.4] vs 1.3 [1.1–1.5], *p* = 0.0178), and in the hilar and mediastinal lymph nodes, suggestive of reactive lymphadenopathy (3.5 [2.7–4.3] vs 1.6 [1.3–2.0], *p* < 0.0001) (Fig. 3).

### Higher [^68^Ga]Pentixafor uptake in patients with pulmonary progression

**Fig. 4:**
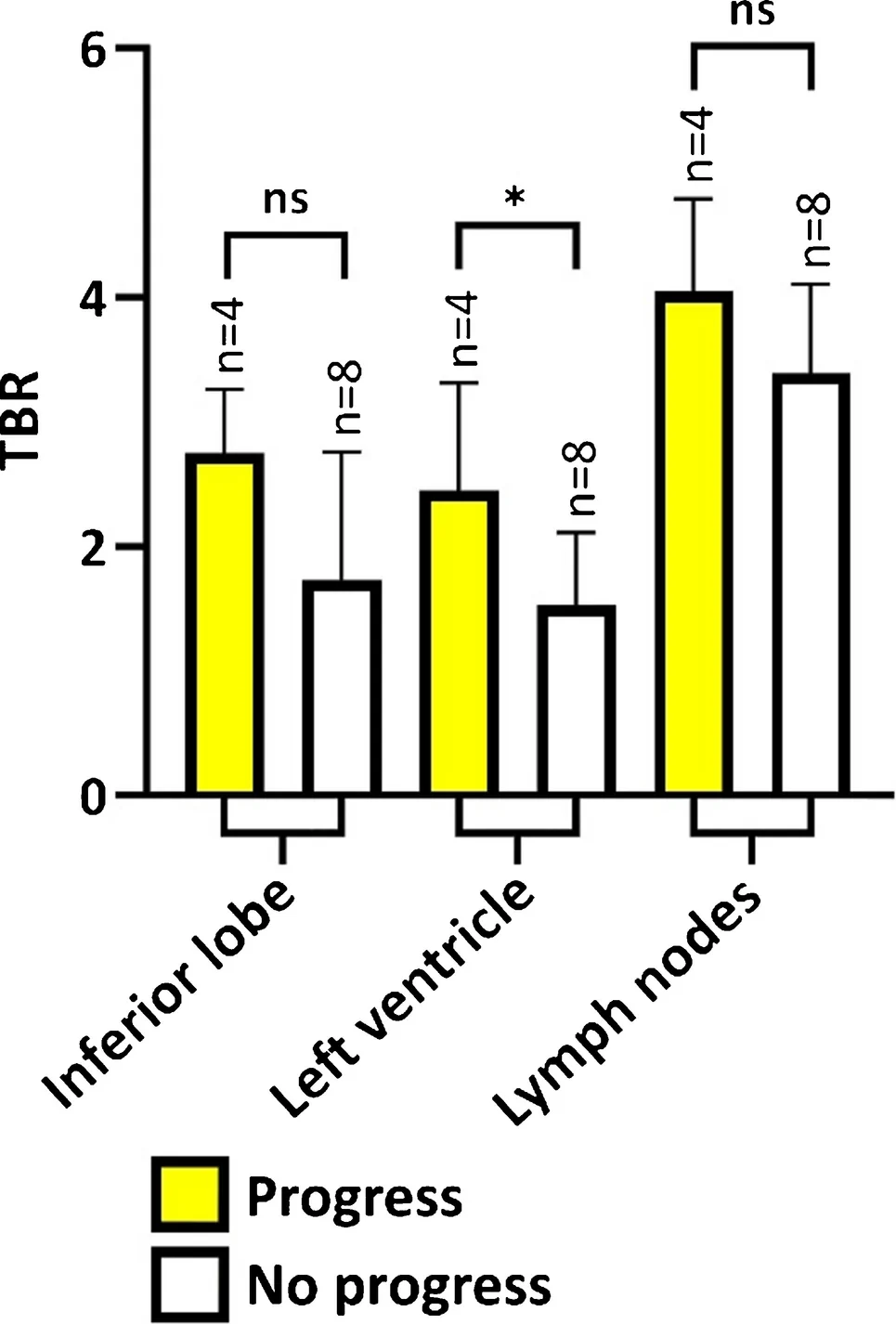
Subgroup analysis of [^68^Ga]Pentixafor uptake in patients with pulmonary progression after CXCR4-PET/CT SSc patients with progressive lung disease (yellow bars) and SSc patients with stable disease/no progression (white bars). *TBR* target-to-background ratio, *ns* not significant

During a median follow-up of 9.2 months, four SSc patients exhibited pulmonary progression and eight patients remained stable. Numerically higher [^68^Ga]Pentixafor uptake in progressive SSc patients compared to patients with stable disease was detected in all three regions. The difference was significant only for the left ventricle. TBR values in progressive versus stable patients were as follows: inferior lung lobes: 2.8 (IQR 2.2–3.3) vs 1.7 (1.1–2.8), *p* = 0.1535; left ventricle: 2.5 (2.1–3.3) vs 1.5 (1.3–2.1), *p* = 0.0283; lymph nodes: 4.1 (3.2–4.8) vs 3.4 (1.9–4.1), *p* = 0.2828. Representative data are shown in Fig. 4. No patient in the SSc cohort exhibited cutaneous progression.

### No relevant side-dependent differences in [^68^Ga]Pentixafor uptake

As an internal plausibility analysis, [^68^Ga]Pentixafor uptake was compared between left- and right-sided pulmonary and lymph node regions to assess potential side-dependent differences or systematic lateralization effects related to VOI placement. No significant differences were observed between the left and right sides (Supplementary Figure S1).

### No correlation between clinical or serological parameters and [^68^Ga]Pentixafor uptake

No significant correlations were found between [^68^Ga]Pentixafor uptake of the whole lung and lung function parameters (FVC and DLCO). Neither was a significant correlation detectable between [^68^Ga]Pentixafor uptake of the inferior lung lobes or the lymph nodes, respectively and ESR, CRP, SDF-1, and mRSS. Finally, no correlation was observed between left ventricular [^68^Ga]Pentixafor uptake and serum troponin (Supplementary Figure S2). Left ventricular TBR did not differ significantly between SSc patients with cardiac involvement (1.3 [IQR 1.3–2.3]; *n* = 3) and those without cardiac involvement (2.1 [1.5–2.5], *p* = 0.3727; *n* = 9).

### No differences in [^68^Ga]Pentixafor uptake across SSc subgroups

Subgroup analyses were performed according to immunosuppressive treatment, Scl-70 autoantibody status, and EUSTAR disease activity index. No significant differences in [^68^Ga]Pentixafor uptake were observed across these subgroups for the inferior lung lobes, left ventricular myocardium, or hilar/mediastinal lymph nodes. Detailed subgroup results are provided in Supplementary Table S2.

## Discussion

This prospective study provides proof-of-concept evidence that CXCR4-targeted PET/CT may visualize biologically active inflammatory and/or remodeling-associated processes in patients with early-stage SSc-ILD. Organs commonly affected by inflammation and consecutive fibrosis in SSc showed a significant increased [^68^Ga]Pentixafor uptake. In particular, [^68^Ga]Pentixafor uptake was higher in inferior lung lobes with NSIP confirmed by CT compared with unaffected superior lobes. HRCT findings were interpreted as reflecting structural lung involvement, which may include both inflammatory and fibrotic components. Additionally, compared with controls, SSc patients exhibited a significantly higher uptake in the inferior lung lobes, in the hilar and mediastinal lymph nodes, and in the left ventricular myocardium.

Conventional imaging in SSc-ILD primarily depicts structural abnormalities but provides only limited information regarding ongoing biological activity within affected tissue. In particular, HRCT cannot reliably distinguish active inflammatory processes from irreversible fibrosis or early fibrogenic remodeling. In this context, CXCR4-targeted molecular imaging may provide complementary information by visualizing CXCR4-associated inflammatory and/or remodeling-related biological activity in vivo. However, the present findings should be interpreted cautiously, as histopathologic validation was not available and tracer uptake cannot be conclusively attributed to inflammatory cell infiltration alone. It therefore remains possible that [^68^Ga]Pentixafor uptake reflects a combination of inflammatory and early fibrogenic remodeling processes.

This interpretation is consistent with recent developments in molecular imaging of fibrotic lung disease, where imaging approaches increasingly aim to capture not only inflammation but also early tissue remodeling. For example, matrix metalloproteinase-2-targeted imaging has recently been proposed as a complementary approach to detect early fibrogenic remodeling in idiopathic pulmonary fibrosis. Together with CXCR4-targeted imaging, such approaches highlight the potential of molecular imaging to characterize biologically active tissue remodeling beyond structural HRCT findings [18].

[^68^Ga]Pentixafor binds to CXCR4, a receptor expressed on several leukocyte subsets^7^, and may therefore serve as an imaging surrogate of CXCR4-associated inflammatory cell recruitment. However, given the lack of histopathologic validation in the present study, tracer uptake should not be interpreted as direct evidence of leukocyte infiltration. The increased tracer uptake observed in CT-defined ILD-affected lung regions, draining pulmonary lymph nodes, and the myocardium supports the feasibility of CXCR4-targeted PET/CT for visualizing biologically active tissue processes in SSc, rather than providing definitive separation of inflammation and fibrosis. We focused on patients with early-stage SSc-ILD to enrich the cohort for potentially biologically active inflammatory and remodeling-associated processes, while acknowledging that imaging alone cannot definitively distinguish active inflammation from fibrotic remodeling. One of the most relevant questions in clinical routine of SSc patients is the differentiation of inflammatory activity (alveolitis) versus (micro)fibrosis. Both entities are hard to differentiate with routine CT scans because both conditions appear as ground-glass opacity [19]. [^68^Ga]Pentixafor-PET/CT may provide additional information on inflammatory activity beyond structural imaging, but differentiation between inflammation and fibrosis cannot be definitively established based on the present data. If future studies demonstrate that CXCR4-PET/CT identifies biologically active disease and patients at risk of progression, increased [^68^Ga]Pentixafor uptake in affected organs could support treatment stratification in selected patients.

CXCR4-imaging is gaining increasing attention, as a group from India published a CXCR4-PET study with an alternative tracer ([^68^Ga]-CPCR4 Trifluoroacetate) in SSc recently^12^. We demonstrated that SSc patients who develop progressive pulmonary disease during follow-up after [^68^Ga]Pentixafor-PET/CT had a higher uptake in the myocardium of the left ventricle compared to patients with stable lung disease. There was also a numerically higher [^68^Ga]Pentixafor uptake in fibrotic lung regions and in the lymph nodes in patients with pulmonary progression, yet without statistical significance presumably due to the small cohort size. A direct causal connection between higher cardiac [^68^Ga]Pentixafor uptake and lung progression is probably not present, but our data indicate that a high [^68^Ga]Pentixafor uptake might correlate with progressive disease in general. The observed associations with pulmonary progression should be interpreted cautiously given the small sample size and exploratory nature of the analysis. GCA patients were chosen as controls, because lung involvement and reactive hilar or mediastinal lymphadenopathy are not characteristic features of GCA. However, GCA is a systemic inflammatory disease, therefore an increased background CXCR4 activity due to immune activation cannot be excluded. A group of healthy controls would not have had this limitation for comparison, but was not available.

Serum troponin levels are often used as a surrogate marker of myocardial involvement in SSc. However, no correlation between serum troponin levels and myocardial [^68^Ga]Pentixafor uptake was observed. Interestingly, tracer uptake was significantly higher in the myocardium of SSc patients compared to controls, although only a subset of patients showed evidence of cardiac involvement based on conventional diagnostics. The increased myocardial uptake observed in SSc patients compared to controls may reflect subclinical inflammatory processes; however, this finding requires further validation and should be interpreted cautiously. The study has several limitations. The sample size was small and the study was conducted at a single center. Furthermore, only low-dose CT was permitted in conjunction with PET due to radiation safety regulations. Future studies might combine [^68^Ga]Pentixafor-PET with lung MRI, as advances in pulmonary MRI may allow improved differentiation between fibrosis and inflammation [20, 21]. Additionally, the role of [^68^Ga]Pentixafor-PET/CT in long-standing SSc with extensive fibrosis warrants further investigations. A head-to-head study comparing [^18^F]FDG-PET/CT and CXCR4-targeted PET/CT should be pursued in the future to investigate if CXCR4-targeted PET/CT is superior.

In conclusion, this study provides initial evidence that CXCR4-targeted PET/CT may visualize biologically active inflammatory and/or remodeling processes in early-stage SSc-ILD. These findings support the feasibility of CXCR4-targeted PET/CT as a complementary molecular imaging approach and highlight the need for validation in larger prospective studies with longitudinal imaging and, where feasible, histopathologic correlation.

## Supplementary Information

Below is the link to the electronic supplementary material.Supplementary file1 (DOCX 585 KB)

## Data Availability

The data that support the findings of this study are not openly available but are available from the corresponding author upon reasonable request.

## Abbreviations

ACR: American college of rheumatology
CRP: C-reactive protein
CT: Computed tomography
CXCR4: C-X-C motif chemokine receptor 4
DLCO: Diffusion capacity for carbon monoxide
EULAR: European alliance of associations for rheumatology
FDG: Fluoro-D-glucose
FVC: Forced vital capacity
GCA: Giant cell arteritis
ILD: Interstitial lung disease
IQR: Interquartile range
mRSS: Modified Rodnan skin score
PET: Positron emission tomography
SSc: Systemic sclerosis
TBR: Target-to-background ratio
