# Supplementary material for: CXCR4-targeted PET/CT in early systemic sclerosis–associated interstitial lung disease: a prospective proof-of-concept study of in vivo inflammatory activity
Source: Eur J Nucl Med Mol Imaging. 2026 Jun 16;53(11):6632–40. doi: 10.1007/s00259-026-08000-3 (PMC13526056; doi:10.1007/s00259-026-08000-3)
Supplement: Supplementary file 1 [file 259_2026_8000_MOESM1_ESM.docx]

**Supplementary Figure S1. Comparison of the [^68^Ga]Pentixafor** **uptake between left and right sides.** [^68^Ga]Pentixafor uptake in systemic sclerosis patients (purple) and in the control group (white). Compared were the left and right side of the superior lung lobe, left and right side of the inferior lung lobe, and left hilar, right hilar, and mediastinal lymph nodes of the lung. A) SUVmax, B) SUVmean, and C) target-to-background ratio (TBR). *ns* not significant

**Supplementary Figure S2. Correlation between the [^68^Ga]Pentixafor** **uptake and clinical and serological parameters.** A) Correlation between the target-to-background ratio (TBR) of [^68^Ga]Pentixafor uptake of the total lung and forced vital capacity (FVC), B) correlation between TBR of the total lung and diffusion capacity for carbon monoxide (DLCO), C) correlation between TBR of the inferior lobes and the modified Rodnan skin score (mRSS), D) correlation between TBR of the lymph nodes and mRSS, E) correlation between TBR of the inferior lobes and the erythrocyte sedimentation rate (ESR), F) correlation between TBR of lymph nodes and ESR, G) correlation between TBR of the inferior lobes and C-reactive protein (CRP), H) correlation between TBR of lymph nodes and CRP, I) correlation between TBR of the inferior lobes and SDF-1 levels in the serum, J) correlation between TBR of lymph nodes and SDF-1 levels, K) correlation between TBR of the left ventricle and troponin values in the serum.

**Supplementary Figure S3. Representative examples of VOI placement on fused [^68^Ga]Pentixafor PET/CT images in one patient.**
(A) Representative placement of standardized 2-cm³ pulmonary VOIs in visually unaffected parenchyma of the left upper lobe (3 posterior + 1 ventral)
(B) Representative placement of standardized 2-cm³ pulmonary VOIs in CT-defined ILD-affected parenchyma of the left basal lower lobe (3 medial posterior + 3 basal posterior.
(C) Representative placement of one background VOI in the superior vena cava for blood-pool reference.
(D) Representative placement of a VOI in a right hilar lymph node (white circle) and 3 VOIs in the left ventricular myocardium (red circles).
Pulmonary VOIs were standardized to 2 cm³ and placed in predefined anatomical regions while avoiding bronchi, pleural structures, and motion-related artifacts. Lymph node and myocardial VOIs were adapted to the respective anatomical structure to avoid spillover from adjacent tissue and blood-pool activity.

| **Statistical region** | **VOIs** | **Number of VOIs** |
| --- | --- | --- |
| Right upper lobe | 3 posterior + 1 ventral | 4 |
| Left upper lobe | 3 posterior + 1 ventral +  1 ventral lingula | 5 |
| Middle lobe | 1 ventral | 1 |
| Right lower lobe | 3 medial posterior +  3 basal posterior | 6 |
| Left lower lobe | 3 medial posterior +  3 basal posterior | 6 |
| Total |  | 22 |

**Supplementary Table S1. Anatomically approximated lobar allocation of the 22 pulmonary VOIs.** The 22 pulmonary VOIs were allocated to statistical lung regions based on an anatomically approximated lobar assignment of the standardized sampling scheme. VOI, volume of interest.

| **Subgroup** | **n** | **Inferior lung lobes TBR, median [IQR]** | **p value** | **Left ventricle TBR, median [IQR]** | **p value** | **Lymph nodes TBR, median [IQR]** | **p value** |
| --- | --- | --- | --- | --- | --- | --- | --- |
| **Immunosuppressive treatment** |  |  |  |  |  |  |  |
| Mycophenolate | 8 | 2.3 [1.7–2.8] | ref. | 2.0 [1.4–2.6] | ref. | 4.1 [3.0–4.3] | ref. |
| Rituximab | 2 | 2.5 [1.7–3.4] | 0.8889 vs MMF | 1.8 [1.4–2.2] | 0.8889 vs MMF | 2.6 [1.6–3.6] | 0.2667 vs MMF |
| No immunosuppression | 2 | 1.7 [0.6–2.9] | 0.8889 vs MMF; 0.6667 vs RTX | 1.5 [0.7–2.4] | 0.7111 vs MMF; >0.9999 vs RTX | 2.3 [1.0–3.7] | 0.2667 vs MMF; >0.9999 vs RTX |
| **Anti-Scl-70 antibody status** |  |  |  |  |  |  |  |
| Scl-70 positive | 8 | 2.1 [1.7–2.8] | ref. | 1.8 [1.4–2.5] | ref. | 3.4 [2.9–4.2] | ref. |
| Scl-70 negative | 4 | 2.5 [1.0–3.3] | 0.6828 | 2.2 [1.0–2.4] | 0.9333 | 3.8 [1.7–4.8] | 0.8081 |
| **EUSTAR disease activity index** |  |  |  |  |  |  |  |
| Active disease | 2 | 2.6 [1.8–3.4] | 0.4848 | 1.7 [1.3–2.1] | 0.6061 | 3.6 [3.2–4.0] | >0.9999 |
| Inactive disease | 10 | 2.3 [1.5–2.8] | ref. | 2.0 [1.4–2.5] | ref. | 3.7 [2.6–4.3] | ref. |

**Supplementary Table S2. Subgroup analyses of [^68^Ga]Pentixafor uptake in SSc patients**

Values are given as median [interquartile range]. TBR was calculated as SUV_max_ of the respective target region divided by SUV_mean_ of the superior vena cava. EUSTAR disease activity was defined as inactive for scores <2.5 and active for scores ≥2.5.ref.,reference group, MMF, mycophenolate; RTX, rituximab; SSc, systemic sclerosis; TBR, target-to-background ratio.
